# Alcohol consumption patterns and adherence to the Mediterranean diet in the adult population of Spain

**DOI:** 10.1007/s00394-023-03318-2

**Published:** 2024-01-13

**Authors:** Julia Fontán-Vela, Cristina Ortiz, Teresa López-Cuadrado, María Téllez-Plaza, Esther García-Esquinas, Iñaki Galán

**Affiliations:** 1 National Centre for Epidemiology, Institute of Health Carlos III, Monforte de Lemos 5, 28029 Madrid, Spain; 2 Department of Preventive Medicine and Public Health, Autonomous University of Madrid, Madrid, Spain; 3 Consortium for Biomedical Research in Epidemiology and Public Health (CIBER en Epidemiología y Salud Pública - CIBERESP), Madrid, Spain

**Keywords:** Alcohol, Consumption patterns, Binge drinking, Alcoholic beverages, Mediterranean diet

## Abstract

**Purpose:**

The objective is to evaluate the association between various indicators of alcohol consumption and the degree of adherence to the Mediterranean diet among the Spanish adult population.

**Methods:**

A cross-sectional study including 44,834 participants ≥ 15 years of age from the 2017 National Health Survey and the 2020 European Health Survey in Spain. Alcohol patterns were defined based on (1) average intake: individuals were classified as low risk (1–20 g/day in men and 1–10 g/day in women) and high risk (> 20 g/day in men or > 10 g/day in women), (2) binge drinking, and (3) alcoholic beverage preference. Non-adherence to the Mediterranean diet was defined as scoring < 7 points on an adapted Mediterranean Diet Adherence Screener index (range 0–10). Odds ratios (OR) were estimated using logistic regression models adjusted for relevant covariates.

**Results:**

Compared to non-drinkers, low and high-risk drinkers were more likely to report non-adherence to the Mediterranean diet: ORs 1.35 (95% CI 1.23; 1.49) and 1.54 (95% CI 1.34; 1.76), respectively. Similarly, reports of binge drinking less than once a month was associated with higher likelihood of non-adherence (OR 1.17; 95% CI 1.04; 1.31). Individuals reporting no preference for a specific beverage and those with a preference for beer or for spirits had lower adherence: ORs 1.18 (95% CI 1.05; 1.33), 1.31 (95% CI 1.17; 1.46), and 1.72 (95% CI 1.17; 2.54), respectively, while a preference for wine showed no association (OR 1.01; 95% CI 0.90; 1.13).

**Conclusion:**

Alcohol consumption, even in low amounts, is associated with lower adherence to the Mediterranean diet. Therefore, alcoholic beverages should not be included in measures that define the Mediterranean diet.

**Supplementary Information:**

The online version contains supplementary material available at 10.1007/s00394-023-03318-2.

## Background

Alcohol consumption is one of the leading causes of death and disability worldwide, with risk increasing with rising levels of consumption [1]. In 2016, excessive alcohol consumption was responsible for 3 million deaths, 5.3% of all deaths, and 5.1% of all Disability-Adjusted Life Years (DALYs) [2]. Further, about 750,000 new cancer cases, 4.1% of the total, were ascribed worldwide to this risk factor in 2020 alone [3].

Alcohol is an intricate component of any diet with numerous effects on appetite [4]. A recent meta-analysis concludes that not only alcohol consumption increases energy intake but that even low amounts of alcohol increase food consumption [5]. Overall consensus is that excessive alcohol drinkers generally have lower diet quality [6–10], with the discussion increasingly focusing on evidence linking light alcohol consumption and poorer diet [6, 10]. However, others fail to detect dietary differences between light-drinkers and non-drinkers [8, 9]. The scientific community´s ongoing debate on the potential benefits of low-to-moderate alcohol consumption on all-cause and cardiovascular disease-mortality [11, 12] only underscores the importance of ascertaining the alcohol-diet association; as any such health benefits may actually be accounted for by drinkers´ dietary habits differing from those of non-drinkers [9, 13].

Many studies on the alcohol-diet relationship analyze how the quantity of alcohol ingested affect diet characteristics. However, consumption patterns may be also associated with variations in diet quality and, thus, examining patterns would contribute to a better understanding of this association [7]. Finally, it is essential to consider the role of binge drinking, as well as the type of alcoholic beverage consumed, since large variations in the results have been described depending on the geographical area [14].

The Mediterranean diet (MD) stands for a dietary pattern characteristic of the European southern regions in the early 1960s and considered a model for a healthy diet [15–17]. Moderate alcohol consumption, especially wine, has traditionally been counted as one of its features [17]. In fact, pictograms describing the MD usually include representations of alcoholic beverages [18]. However, previous studies conducted in Spain [19] cast doubt on this relationship, concluding that alcohol is currently not one of the MD´s distinctive elements.

This study´s main aim was to evaluate the association between various alcohol consumption patterns and the adherence level to the MD among a representative sample of the Spanish adult population.

## Methods

### Study design and population

We analyzed data from two surveys, the 2017 Spanish National Health Survey and the 2020 European Health Survey in Spain. These surveys are representative of the Spanish population and use harmonized sample design and questionnaires collecting data on sociodemographic characteristics, health status, social and environmental determinants, lifestyles, and access to and use of health services. Their multistage sampling selects all provinces first, followed by a sample of municipalities within each province, stratified by population size. Subsequently, a sample of census tracts is selected within these municipalities. Finally, a sample of households is picked within each tract, and an adult aged ≥ 15 years is chosen. Participants are interviewed face-to-face with the aid of an electronic device (Computer-Assisted Personal Interviewing-CAPI) [20, 21].

The overall response rate (number of completed interviews relative to the total number of eligible households) was 70.3%, resulting in an initial sample size of 45,161 subjects. After excluding participants with missing data in any of the study variables, the final sample consisted of 44,834 individuals. In the case of 1417 participants, the survey was answered by a proxy (a person responding on behalf of the selected respondent).

### Study variables

#### Alcohol consumption patterns

Alcohol consumption (frequency and amount) of six types of alcoholic beverages (wine, beer, vermouth and aperitifs, spirits, mixed drinks, and cider) was recorded and analyzed. Participants were classified into the following categories: non-drinkers (individuals who never drank alcohol or only took a few sips throughout their life), former drinkers (individuals who reported quitting alcohol and reported zero consumption in the past 12 months), occasional drinkers (≤ 1 time per week), low-risk alcohol consumers (up to 20 g/day for men and 10 g/day for women) and high-risk alcohol consumers (> 20 g/day for men and > 10 g/day for women) [22]. Binge drinking was defined as the consumption of ≥ 6 or ≥ 5 standard drinks within a 4–6 h period for men and women, respectively [23], and its frequency classified as never, < 1 time per month, monthly, weekly/daily. Alcoholic beverage preference for wine, beer, or spirits was considered defined if the consumption of any of these beverages accounted for ≥ 80% of the total alcohol intake [19, 24]. Additionally, we estimated total consumption of each beverage in grams per day (0, > 0–10, > 10–20, > 20).

#### Mediterranean diet

We used an adapted version of the Mediterranean Diet Adherence Screener (MEDAS) [25] to measure participants’ degree of adherence to the MD. We assigned a score ranging from 0 to 10 based on the reported consumption of the following items: 1–2 servings of fruit per day (1 point), > 3 servings per day (2 points); 1 serving of vegetables per day (1 point), > 1 serving per day (2 points); ≥ 3 servings of legumes per week (1 point); ≥ 3 servings of fish per week (1 point); < 1 serving of meat per day (1 point); < 1 serving of sugary drinks per day (1 point); < 3 servings of sweets per week (1 point); < 3 servings of fast food (including snacks) per week (1 point). A score < 7 points was considered non-adherence to the MD.

#### Covariates

The following variables were included in multivariate analyses as covariates: Age group (15–24 years of age, 25–34, 35–44, 45–54, 55–64, 65–74, and ≥ 75); sex; educational level [primary education (6–12 years of schooling) or lower, first stage of secondary education (12–16 years of schooling), second stage of secondary education (16–18 years of schooling), university education (≥ 18 years of schooling)]; country of birth (Spain, other); size of residential municipality (> 500,000 inhabitants, province capital, > 100,000–500,000, > 50,000–100,000, > 20,000–50,000, 10,000–20,000, and < 10,000); body mass index (BMI) [ratio of weight (kg) to square of height (m)]; tobacco consumption (non-smoker, former smoker, smoker of up to 14 cigarettes/day, smoker > 14 cigarettes/day); sedentary leisure time (some degree of physical activity or none); perceived health status (very good, good, fair, poor, very poor); and survey year (2017, 2020).

### Statistical analysis

We conducted descriptive analyses to ascertain the sample´s prevalence and distribution of sociodemographic characteristics, lifestyle factors, perceived health status, and MD adherence by alcohol consumption pattern. Associations with non-adherence to the MD were analyzed with fully-adjusted multivariate logistic regression models, estimating odds ratios (OR) with their respective 95% confidence intervals (CI). Binge drinking was also adjusted for when analyzing average alcohol consumption and, vice versa, we controlled for quantity of alcohol consumed when evaluating binge drinking. Further, in the models where the primary independent variable was alcoholic beverage preference, we took into account both total daily alcohol intake (g/day) and binge drinking. Finally, when wine, beer, and liquor consumption (0 g/day, > 0–10, > 10–20, and > 20) were the primary independent variable, the models were adjusted for binge drinking.

Furthermore, we assessed whether these associations varied by age group (< 65 vs ≥ 65), sex, or educational level (4 categories) including the corresponding interaction terms in the models. When any interaction terms were statistically significant (p < 0.05), the results were stratified according to the corresponding sociodemographic variables.

Sensitivity analyses included examining these associations using the MEDAS adapted index in a continuous form. First, the Spearman’s correlation between the continuous form of the MEDAS index and the alcohol total consumption (g/day) was calculated. Second, coefficients β were estimated through linear regression models adjusted for the same covariates. Third, logistic regression models of the associations of interest were also repeated excluding proxies´ data. Finally, logistic regression models examining binge drinking and total consumption of each type of beverage regarding MD non-adherence were repeated excluding former drinkers.

The estimates were weighted using sampling weights to ensure proportional representation. All analyses were conducted using the survey procedure in Stata v.17 (StataCorp, College Station, USA) to incorporate the complex sample design features of the surveys.

## Results

Tables 1 and 2 show the characteristics of the sample according to alcohol consumption patterns. Among the participants, 21.5% were classified as non-drinkers, 13% as former drinkers, 29.7% as occasional drinkers, 28.1% as low-risk drinkers, and 7.7% as high-risk drinkers. Binge drinking in the previous 30 days was reported by 7% of the participants. Among drinkers, 21.2%, 35.7%, and 2.8% reported preference for wine, beer, and spirits, respectively. A vast majority of the study sample, 76.3%, reported no adherence to the MD.

**Table 1:** Characteristics of the study sample according to average alcohol consumption

		Average alcohol consumption				
	N	Non-drinkers	Former drinkers	Occasional drinkers	Low risk	High risk
N	44,834	9470	6472	12,768	12,416	3708
Sex						
Men	20,814	29.5	43.4	45.2	66.4	58.7
Women	24,020	70.6	56.6	54.8	33.6	41.3
Age (years)	44,834	48.9 (22.1)	55.7 (20.5)	44.2 (17.2)	49.6 (16.5)	50.9 (16.8)
Country of birth						
Spain	40,592	78.7	89.1	85.8	88.6	88.9
Other country	4242	21.3	10.9	14.2	11.4	11.1
Size of municipality (population)						
Rural (< 10,000)	10,172	19.6	24.3	19.0	19.7	26.4
Urban (≥ 10,000)	34,662	80.4	75.7	81.0	80.3	73.6
Educational level						
Primary or less	13,408	38.8	37.6	16.4	18.0	21.4
1st stage secondary	10,786	28.0	26.5	26.8	21.9	27.0
2nd stage secondary	12,237	22.0	23.1	35.3	33.7	30.7
University/college	8403	11.2	12.8	21.5	26.4	20.9
BMI (kg/m^2^)						
Underweight	877	3.4	1.8	2.8	1.4	1.9
Normal weight	18,148	43.2	36.5	47.9	41.9	40.6
Overweight	16,399	30.7	36.1	32.2	40.2	38.9
Obesity	7246	16.5	19.9	13.7	14.5	17.0
No response	2164	6.2	5.8	3.3	2.0	1.6
Tobacco consumption						
Non smoker	23,580	78.1	53.0	54.1	40.4	28.6
Former smoker	11,277	10.6	26.8	21.3	32.0	31.3
Smoker 1–14 cigs/day	6535	7.8	13.2	17.7	18.7	22.5
Smoker > 14 cigs/day	3442	3.5	7.0	6.9	8.9	17.6
Leisure time sedentarism						
No	27,913	55.1	52.0	65.4	70.9	64.3
Yes	16,921	44.9	48.0	34.6	29.1	35.7
Perceived health status						
Very good	8871	24.0	14.1	28.0	24.2	19.0
Good	21,840	42.2	42.5	50.2	55.0	59.0
Neither good nor poor	10,051	22.9	26.8	16.7	16.4	16.9
Poor	3190	8.3	12.6	4.1	3.5	4.2
Very poor	882	2.6	4.0	1.0	0.9	0.9
Survey year						
2017	22,954	47.4	52.9	48.4	49.5	54.0
2020	21,880	52.6	47.1	51.6	50.5	46.0
Adherence to Mediterranean diet						
No^a^	33,581	73.0	74.4	77.0	78.0	80.3
Yes	11,253	27.0	25.6	23.0	22.0	19.7

The values presented here are percentages and weighted averages with their corresponding standard deviation in parentheses. The “N” are unweighted values

^a^Score < 7 points in the adapted MEDAS index (0–10 points)

**Table 2:** Characteristics of the study sample according to alcohol consumption patterns

		Alcohol consumption patterns					
		Binge drinking		Beverage preference			
	*N*	No	Yes	No preference	Wine	Beer	Spirits
N	44,834	42,033	2801	6431	4158	5165	370
Sex							
Men	20,814	47.0	70.4	69.7	56.6	64.2	62.4
Women	24,020	53.0	29.6	30.3	43.4	35.8	37.6
Age (years)	44,834	49.4 (19.1)	39.5 (14.7)	48.1 (15.8)	62.5 (16.8)	45.7 (13.2)	34.5 (15.0)
Country of birth							
Spain	40,592	85.7	86.5	88.7	90.6	87.5	86.5
Other country	4242	14.3	13.5	11.3	9.4	12.5	13.5
Size of municipality (population)							
Rural (< 10,000)	10,172	20.6	20.5	21.5	26.9	17.3	21.1
Urban (≥ 10,000)	34,662	79.4	79.5	78.5	73.1	82.7	78.9
Educational level							
Primary or less	13,408	25.7	22.7	15.0	34.7	13.8	12.7
1st Stage Secondary	10,786	25.6	37.3	22.9	17.0	25.6	37.4
2nd Stage Secondary	12,237	29.5	26.8	34.3	26.1	35.8	33.9
University/College	8403	19.2	13.2	27.8	22.2	24.8	16.0
BMI (kg/m^2^)							
Underweight	877	2.3	2.2	1.3	1.4	1.6	3.1
Normal weight	18,148	42.8	47.3	40.8	36.6	44.7	51.7
Overweight	16,399	35.2	35.2	41.1	41.5	38.6	29.0
Obesity	7246	15.7	14.2	15.6	17.3	13.3	11.6
No response	2164	4.0	1.1	1.2	3.2	1.8	4.6
Tobacco consumption							
Non Smoker	23,580	54.7	34.9	36.2	44.2	35.8	40.2
Former smoker	11,277	23.6	21.7	31.4	40.0	28.6	17.2
Smoker 1–14 cigs/day	6535	14.7	28.7	21.7	10.9	21.6	29.6
Smoker > 14 cigs/day	3442	7.0	14.7	10.7	4.9	14.0	13.0
Leisure time sedentarism							
No	27,913	62.5	68.1	72.6	69.4	66.0	69.7
Yes	16,921	37.5	31.9	27.4	30.6	34.0	30.3
Perceived health status							
Very good	8871	23.0	31.2	23.7	15.0	26.4	33.3
Good	21,840	49.3	51.6	57.8	52.2	56.3	51.9
Neither good nor poor	10,051	19.7	13.3	14.8	24.5	14.0	13.0
Poor	3190	6.2	3.0	3.0	6.7	2.6	1.3
Very poor	882	1.8	0.9	0.7	1.6	0.7	0.5
Survey year							
2017	22,954	49.0	56.7	49.4	52.5	50.1	55.6
2020	21,880	51.0	43.3	50.6	47.5	49.9	44.4
Adherence to Mediterranean diet							
No^a^	33,581	75.8	82.9	79.5	68.6	82.2	89.9
Yes	11,253	24.2	17.1	20.5	31.4	17.8	10.1

The values presented here are percentages and weighted averages with their corresponding standard deviation in parentheses. The “N” are unweighted values

^a^Score < 7 points in the adapted MEDAS index (0–10 points)

### Average alcohol consumption, binge drinking, and Mediterranean diet

Compared to non-drinkers, former and occasional drinkers were more likely to report poorer adherence to the MD (OR 1.18; 95% CI 1.06; 1.30 and OR 1.13; 95% CI 1.03; 1.24, respectively). Adherence to MD was even poorer among low-risk and high-risk drinkers, with ORs of 1.35 (95% CI 1.23; 1.49) and 1.54 (95% CI 1.34; 1.76), respectively (Table 3). No significant differences were observed between individuals who did and did not engage in binge drinking in the previous 30 days (OR 0.90; 95% CI 0.78; 1.03). However, individuals who binge drank less than once a month had lower adherence to the MD (OR 1.17; 95% CI 1.04; 1.31) compared to those who reported no binge drinking at all.

**Table 3:** Association between average alcohol consumption and binge drinking, and non-adherence to the Mediterranean diet

		Non-adherence to the Mediterranean diet	
		Prevalence (%) (95% CI)	OR (95% CI)
Average alcohol consumption^a^			
Non-drinkers	9470	73.0 (71.6; 74.3)	Ref.
Former drinkers	6472	74.4 (73.0; 75.8)	1.18 (1.06; 1.30)
Occasional drinkers	12,768	77.0 (76.0; 78.0)	1.13 (1.03; 1.24)
Low risk	12,416	78.0 (77.0; 78.9)	1.35 (1.23; 1.49)
High risk	3708	80.3 (78.6; 81.9)	1.54 (1.34; 1.76)
Binge drinking^b^			
In the previous 30 days			
No	42,033	75.8 (75.2; 76.5)	Ref.
Yes	2801	82.9 (81.1; 84.6)	0.90 (0.78; 1.03)
Frequency			
Never	37,617	74.7 (74.0; 75.4)	Ref.
< once per month	4416	84.2 (82.9; 85.5)	1.17 (1.04; 1.31)
Monthly	1991	83.2 (81.0; 85.1)	0.96 (0.81; 1.13)
Weekly	810	82.3 (78.9; 85.3)	0.84 (0.66; 1.07)

^a^Logistic regression model adjusted for sex, age, level of education, size of municipality, country of birth, tobacco consumption, leisure time sedentarism, BMI, perceived health status, survey year, and binge drinking

^b^Logistic regression model adjusted for sex, age, level of education, size of municipality, country of birth, tobacco consumption, leisure time sedentarism, BMI, perceived health status, survey year, and total daily alcohol intake (g/day)

### Type of alcoholic beverage and Mediterranean diet

Compared to non-drinkers, former and occasional drinkers and also individuals who reported no preference for a certain alcoholic beverage showed lower adherence to the MD (OR 1.18; 95% CI 1.07; 1.31; OR 1.14; 95% CI 1.04; 1.25, and OR 1.29; 95% CI 1.13; 1.48, respectively) (Table 4). Individuals with a preference for beer reported poorer adherence to the MD (OR 1.43; 95% CI 1.26; 1.63), and the lowest adherence was observed among those with a preference for spirits (OR 1.88; 95% CI 1.26; 2.79). A preference for wine failed to show an association with MD adherence (OR 1.09; 95% CI 0.96; 1.24). The direction of the associations was the same when considering the total consumption of wine, beer, and spirits in a categorized manner (Table 4).

**Table 4:** Association between beverage preference and type of alcohol consumed, and non-adherence to the Mediterranean diet

		Non-adherence to the Mediterranean diet	
		Prevalence (%) (95% CI)	OR (95% CI)
Alcoholic beverage preference^a^			
Non-drinkers	9470	73.0 (71.6; 74.3)	Ref.
Former drinkers	6472	74.4 (73.0; 75.8)	1.18 (1.07; 1.31)
Occasional drinkers	12,768	77.0 (76.0; 78.0)	1.14 (1.04; 1.25)
No preference	6431	79.5 (78.2; 80.8)	1.29 (1.13; 1.48)
Wine	4158	68.6 (66.7; 70.4)	1.09 (0.96; 1.24)
Beer	5165	82.2 (80.9; 83.5)	1.43 (1.26; 1.63)
Spirits	370	89.9 (86.0; 92.8)	1.88 (1.26; 2.79)
Type of alcoholic beverage^b^			
Wine			
0	35,330	76.9 (76.2; 77.6)	Ref.
> 0-10 g/day	7703	73.4 (72.1; 74.6)	0.95 (0.87; 1.03)
> 10-20 g/day	1428	76.1 (73.3; 78.7)	1.17 (1.00; 1.37)
> 20 g/day	373	72.4 (66.2; 77.8)	0.98 (0.73; 1.33)
Beer			
0	33,590	74.6 (73.9; 75.4)	Ref.
> 0-10 g/day	8866	79.6 (78.5; 80.7)	1.26 (1.16; 1.37)
> 10-20 g/day	1811	84.7 (82.4; 86.8)	1.41 (1.18; 1.68)
> 20 g/day	567	90.1 (86.9; 92.5)	2.06 (1.46; 2.89)
Spirits			
0	41,869	75.6 (74.9; 76.2)	Ref.
> 0-10 g/day	2213	85.1 (83.3; 86.8)	1.20 (1.04; 1.40)
> 10-20 g/day	618	86.5 (82.6; 89.7)	1.17 (0.85; 1.61)
> 20 g/day	134	91.8 (85.6; 95.4)	1.75 (0.91; 3.53)

^a^Logistic regression model adjusted for sex, age, level of education, size of municipality, country of birth, tobacco consumption, leisure time sedentarism, BMI, perceived health status, survey year, total daily alcohol intake (g/day), and binge drinking

^b^Logistic regression model adjusted for sex, age, level of education, size of municipality, country of birth, tobacco consumption, leisure time sedentarism, BMI, perceived health status, survey year, alcohol intake (g(day) from other types of alcoholic beverages, and binge drinking

### Subgroup analysis

*Sex*. Statistically significant interactions were observed between sex and low-risk and high-risk consumption, as well as binge drinking less than once a month (p for interaction < 0.05) (Table 5). Men had an OR of 1.50 (95% CI 1.27; 1.77) for low-risk consumption and 1.84 (95% CI 1.49; 2.28) for high-risk consumption, whereas these values were lower in women (OR 1.31; 95% CI 1.16; 1.48; and OR 1.42; 95% CI 1.19; 1.70, respectively). Regarding binge drinking less frequently than once a month, the OR for men was 1.25 (95% CI 1.07; 1.45), and no association was found in women.

**Table 5:** Association between alcohol consumption patterns and non-adherence to the Mediterranean diet, by sex

	Non-Adherence to the Mediterranean diet				
	N	Men OR (95% CI)N		Women OR (95% CI)	p for interaction
Average alcohol consumption^a^					
Non-drinkers	2314	Ref.	7156	Ref.	
Former drinkers	2703	1,40 (1.16; 1.68)	3769	1.12 (1.00; 1.26)	0.072
Occasional drinkers	5479	1.22 (1.04; 1.43)	7289	1.14 (1.02; 1.26)	0.122
Low risk	8118	1.50 (1.27; 1.77)	4298	1.31 (1.16; 1.48)	0.019
High risk	2200	1.84 (1.49; 2.28)	1508	1.42 (1.19; 1.70)	0.006
Binge drinking^b^					
In the previous 30 days					
No	18,850	Ref.	23,183	Ref.	
Yes	1964	0.87 (0.74; 1.04)	837	0.87 (0.70; 1.09)	0.325
Frequency					
Never	15,939	Ref.	21,678	Ref.	
< once per month	2911	1.25 (1.07; 1.45)	1505	1.01 (0.85; 1.19)	0.007
Monthly	1333	0.96 (0.79; 1.18)	658	0.89 (0.69; 1.16)	0.252
Weekly	631	0.83 (0.62; 1.11)	179	0.78 (0.51; 1.20)	0.448
Alcoholic beverage preference^c^					
Non-drinkers	2314	Ref.	7156	Ref.	
Former drinkers	2703	1.39 (1.16; 1.67)	3769	1.12 (1.00; 1.26)	0.082
Occasional drinkers	5479	1.22 (1.04; 1.43)	7289	1.15 (1.03; 1.28)	0.131
No preference	4446	1.37 (1.12; 1.68)	1985	1.31 (1.07; 1.62)	0.083
Wine	2403	1.26 (1.03; 1.54)	1755	1.06 (0.88; 1.28)	0.088
Beer	3233	1.49 (1.21; 1.83)	1932	1.46 (1.23; 1.75)	0.156
Spirits	236	1.97 (1.18; 3.30)	134	1.89 (1.01; 3.55)	0.639

^a^Logistic regression model adjusted for sex, age, level of education, size of municipality, country of birth, tobacco consumption, leisure time sedentarism, BMI, perceived health status, survey year, and binge drinking

^b^Logistic regression model adjusted for sex, age, level of education, size of municipality, country of birth, tobacco consumption, leisure time sedentarism, BMI, perceived health status, survey year, and total daily alcohol intake (g/day)

^c^Logistic regression model adjusted for sex, age, level of education, size of municipality, country of birth, tobacco consumption, leisure time sedentarism, BMI, perceived health status, survey year, total daily alcohol intake (g/day) and binge drinking

*Age*. Statistically significant interactions were observed between age and occasional, low-risk and high-risk consumption, binge drinking and preferred beverage consumption (p for interaction < 0.05). The results of the age-stratified analyses (< 65 vs. ≥ 65) are described in Table 6. Regarding average consumption, the association in both groups were in the same direction but the magnitude was greater in individuals younger than 65 years of age. Regarding binge drinking, the better adherence to the MD among ≥ 65 year-old weekly binge drinkers is noteworthy (OR 0.57; 95% CI 0.39; 0.84). Lastly, individuals under 65 reporting no beverage preference or with a preference for beer and spirits showed poorer adherence to the MD. No association was found among individuals 65 and over.

**Table 6:** Association between alcohol consumption patterns and non-adherence to the Mediterranean diet, by age (< 65 vs ≥ 65)

	N	Non-adherence to the Mediterranean diet			
		< 65 OR (95% CI)		≥ 65 OR (95% CI)	p for interaction
Average alcohol consumption^a^					
Non-drinkers	5546	Ref.	3924	Ref.	
Former drinkers	3484	1.23 (1.07; 1.41)	2988	1.15 (1.01; 1.32)	0.426
Occasional drinkers	10,115	1.19 (1.07; 1.33)	2653	1.10 (0.96; 1.26)	0.009
Low risk	9014	1.48 (1.31; 1.67)	3402	1.20 (1.04; 1.39)	< 0.001
High risk	2615	1.61 (1.36; 1.91)	1093	1.54 (1.26; 1.90)	0.021
Binge drinking^b^					
In the previous 30 days					
No	28,284	Ref.	13,749	Ref.	
Yes	2490	0.90 (0.77; 1.05)	311	0.66 (0.50; 0.87)	0.002
Frequency					
Never	24,233	Ref	13,384	Ref	
< once per month	4051	1.14 (1.01; 1.29)	365	1.06 (0.79; 1.41)	0.065
Monthly	1838	0.93 (0.78; 1.11)	153	0.80 (0.53; 1.20)	0.146
Weekly	652	0.93 (0.69; 1.24)	158	0.57 (0.39; 0.84)	0.004
Alcoholic beverage preference^c^					
Non-drinkers	5546	Ref.	3924	Ref.	
Former drinkers	3484	1.23 (1.07; 1.41)	2988	1.16 (1.01; 1.33)	0.456
Occasional drinkers	10,115	1.20 (1.07; 1.34)	2653	1.10 (0.96; 1.27)	0.009
No preference	5004	1.40 (1.19; 1.65)	1427	1.05 (0.84; 1.32)	< 0.001
Wine	1806	1.09 (0.91; 1.30)	2352	1.11 (0.93; 1.32)	0.786
Beer	4495	1.52 (1.31; 1.76)	670	1.22 (0.96; 1.54)	0.001
Spirits	324	2.15 (1.34; 3.44)	46	0.88 (0.41; 1.90)	0.005

^a^Logistic regression model adjusted for sex, age, level of education, size of municipality, country of birth, tobacco consumption, leisure time sedentarism, BMI, perceived health status, survey year, and binge drinking

^b^Logistic regression model adjusted for sex, age, level of education, size of municipality, country of birth, tobacco consumption, leisure time sedentarism, BMI, perceived health status, survey year, and total daily alcohol intake (g/day)

^c^Logistic regression model adjusted for sex, age, level of education, size of municipality, country of birth, tobacco consumption, leisure time sedentarism, BMI, perceived health status, survey year, total daily alcohol intake (g/day) and binge drinking

*Educational level*. No statistically significant interactions were detected between educational achievement and alcohol consumption indicators (data not shown).

### Sensitivity analysis

The Spearman’s correlation coefficient between the continuous form of the MEDAS index and the alcohol total consumption (g/day) was − 0.06. When compared to non-drinkers, linear regression results show that any drinker, regardless of the amount of average alcohol reported, scored a significantly lower adherence to the MD (measured with the MEDAS index in its continuous form). This was even more notable in low-risk and high-risk drinkers (Supplementary Table 1). Further regression analyses on the type of beverage produced similar results to those observed using the categories of the MEDAS index (Supplementary Table 2).

Overall analyses excluding proxies, as well as analyses of binge drinking and beverage preference excluding former drinkers, produced similar results to those observed for the entire sample (Supplementary Table 3, 4, and 5).

## Discussion

The main results of the study show that, compared to the abstemious population, both low-risk and high-risk alcohol consumption are associated with poorer adherence to the MD. This association is also observed among former and occasional drinkers, occasional binge drinkers, those with no beverage preference as well as among those with a preference for beer or for spirits.

Our findings support past work concluding that both low-risk and high-risk alcohol consumers had diets of poorer quality compared to abstainers or non-drinkers [7, 10]. Overall, poorer diet quality has been reported as average alcohol consumption increases [7], as our results confirm regarding adherence to the MD. However, other authors [8, 9, 24] failed to detect any significant differences between the dietary patterns of moderate drinkers and non-drinkers.

It is worth highlighting the following work conducted with similar methods as ours. León-Muñoz and colleagues, using population-based data aged 18–64 years from Spain, also failed to find differences in adherence to the MD between moderate drinkers and non-drinkers. However, adherence was significantly lower in high-risk drinkers [19]. These findings, in turn, supported Valencia-Martín et al.’s previous study in Madrid region [24] regarding poor diet and excessive drinking.

Regarding gender differences, men´s greater magnitude of non-adherence to the MD in all categories of average consumption may be related to their higher frequency and quantity of alcohol consumption compared to women’s [26].

In Anglo-Saxon countries, binge drinking tends to be a sporadic practice and, on such occasions, the consumption takes place over a short period of time. In Mediterranean regions, however, the binging tends to occur more often but spread over several hours with a strong social component [23]. Whereas the Anglo-Saxon habit is clearly associated with poorer diet quality [8], findings in Spain are inconclusive. Authors have reported both higher adherence to the MD [19] and poorer adherence to healthy eating guidelines [24] among binge drinkers. Our results contribute to the current controversy because, contrary to expectations, we failed to detect any significant differences in adherence to the MD between binge drinkers and never binge drinkers. Further, only those who binge drank less than once a month reported poorer adherence to the MD than those never engaging in it.

Due to the scarce evidence available, any gender and age differences found in this association were interpreted cautiously. Several studies report higher prevalence of binge drinking in men [26–29], which could be attributed to a combination of biological and cultural factors [26, 27] which, in turn, may also influence the gender-based relationship between binge drinking and diet quality. Meanwhile, compared to their older counterparts (≥ 65), younger individuals are increasingly departing from the MD [30] and they often engage in sporadic binge drinking during nighttime social events or open-air drinking parties known in Spain as “botellón” [31–33]. Binge drinking in older individuals is more often associated to higher frequency and around meals. However, further research should delve into these findings as they may have significant implications for public health.

Regarding beverage preference, our findings support previous descriptions of poorer dietary habits among individuals with a preference for beer or spirits [14, 34]. However, we failed to find any association with a preference for wine consumption as did León-Muñoz and colleagues, who reported a higher adherence to the MD among individuals with a preference for wine compared to those with no beverage preference [19]. We get similar results when excluding non-drinkers from the analysis and using those with no preference as reference category. Another study conducted in Spain reported no association between beverage preference and adherence to the MD [35]. Only in Northern European countries and the United States has a preference for wine consumption been associated with higher-quality dietary patterns [36–38]. This could be attributed to the fact that in Mediterranean countries, wine is consumed across all socioeconomic strata due to its affordable price and strong connection to food consumption.

Among the limitations of this study to consider when interpreting its findings is its cross-sectional nature, which prevents establishing causal associations between alcohol consumption and adherence to the MD. Additionally, the use of self-reported data introduces the possibility of recall bias and underestimation of alcohol consumption, especially for high-risk or occasional consumption [39, 40].

Among the many strengths of the study, we would highlight the comprehensive assessment of alcohol consumption patterns in a large, population-based sample of the Spanish adult population. Particularly relevant was the distinction made among abstainers, former drinkers, and occasional drinkers. These consumer groups often fail to account for high percentages of study samples and are usually combined into a single category as “non-drinkers,” making any interpretation problematic.

Regarding data analysis, a key strength was the full adjustment for relevant covariates in the statistical analyses. Furthermore, sensitivity analyses using the continuous form of MEDAS index confirmed that our results were not due to the cutoff point chosen to define adherence to the MD. Instead, they showed consistent trends supporting the logistic regression results. Lastly, the analyses excluding former drinkers and proxies provided further evidence that the results were robust and consistent across the entire sample.

## Conclusions

Our results challenge the traditional belief that consuming small amounts of alcohol or certain alcoholic beverages, especially wine, are part of the Mediterranean diet pattern. Further prospective studies are needed to confirm these findings and provide more robust evidence on the association between alcohol consumption and dietary patterns. If these findings were confirmed by future studies, it would be advisable to exclude the inclusion of alcohol consumption in dietary quality questionnaires, especially those focused on evaluating adherence to the Mediterranean diet. Furthermore, it may be necessary to emphasize the potential negative effects of alcohol consumption on overall diet quality and health outcomes.

### Supplementary Information

Below is the link to the electronic supplementary material.

Supplementary file1 (DOCX 23 kb)

## Data Availability

The datasets used and/or analysed during the current study may be available from the corresponding author on reasonable request.

## Abbreviations

DALYs: Disability-adjusted life years
MD: Mediterranean diet
MEDAS: Mediterranean diet adherence screener
BMI: Body Mass Index
OR: Odds ratio
CI: Confidence intervals
