# Supplementary material for: Alcohol consumption patterns and adherence to the Mediterranean diet in the adult population of Spain
Source: Eur J Nutr. 2024 Jan 13;63(3):881–91. doi: 10.1007/s00394-023-03318-2 (PMC10948540; doi:10.1007/s00394-023-03318-2)
Supplement: Supplementary file 1 — Supplementary file1 (DOCX 23 kb) [file 394_2023_3318_MOESM1_ESM.docx]

**Annex I**

**Supplementary Table 1.** **Association between average alcohol consumption and binge drinking and non-adherence to the Mediterranean diet (continuous variable of the adapted MEDAS index).**

|  |  | **Non-Adherence to Mediterranean Diet** | |
| --- | --- | --- | --- |
|  | **N** | **Mean**  **(Standard Deviation)** | **β Coefficient**  **(95%CI)** |
|  |  |  |  |
| **Average alcohol consumption^a^** |  |  |  |
| Non-drinkers | 9470 | 5.2 (1.9) | Ref. |
| Former drinkers | 6472 | 5.2 (2.0) | -0.13 (-0.20; -0.05) |
| Occasional drinkers | 12768 | 4.9 (1.9) | -0.13 (-0.20; -0.07) |
| Low risk | 12416 | 5.0 (1.9) | -0.26 (-0.33; -0.19) |
| High risk | 3708 | 4.8 (2.0) | -0.41 (-0.51; -0.31) |
|  |  |  |  |
| ***Binge drinking*^b^** |  |  |  |
| **In the previous 30 days** |  |  |  |
| No | 42033 | 5.1 (1.9) | Ref. |
| Yes | 2801 | 4.4 (1.9) | -0.04 (-0.14; 0.06) |
|  |  |  |  |
| **Frequency** |  |  |  |
| Never | 37617 | 5.2 (1.9) | Ref. |
| <once a month | 4416 | 4.5 (1.9) | -0.16 (-0.23; -0.08) |
| Monthly | 1991 | 4.4 (1.9) | -0.10 (-0.22; 0.02) |
| Weekly | 810 | 4.4 (2.0) | -0.02 (-0.18; 0.14) |

^a^ Linear regression model adjusted for sex, age, level of education, size of municipality, country of birth, tobacco consumption, leisure time sedentarism, BMI, perceived health status, survey year, and binge drinking.

^b^ Linear regression model adjusted for sex, age, level of education, size of municipality, country of birth, tobacco consumption, leisure time sedentarism, BMI, perceived health status, survey year, and total daily alcohol intake (g/day).

**Supplementary Table 2. Association between preference and type of alcoholic beverage and non-adherence to the Mediterranean diet (continuous variable of the adapted MEDAS index).**

|  |  | **Non-Adherence to the Mediterranean Diet** | |
| --- | --- | --- | --- |
|  | **N** | **Mean**  **(Standard Deviation)** | **β Coefficient**  **(95%CI)** |
|  |  |  |  |
| **Alcoholic beverage preference^a^** |  |  |  |
| Non-drinkers^b^ | 9470 | 5.2 (1.9) | Ref. |
| Former drinkers | 6472 | 5.2 (2.0) | -0.13 (-0.20; -0.05) |
| Occasional drinkers | 12768 | 4.9 (1.9) | -0.14 (-0.21; -0.08) |
| No preference | 6431 | 4.8 (1.9) | -0.22 (-0.32; -0.12) |
| Wine | 4158 | 5.6 (1.9) | -0.06 (-0.15; 0.04) |
| Beer | 5165 | 4.7 (1.8) | -0.28 (-0.37; -0.19) |
| Spirits | 370 | 3.7 (1.8) | -0.69 (-0.92; -0.46) |
|  |  |  |  |
| **Type of alcoholic beverage^b^** |  |  |  |
| **Wine** |  |  |  |
| 0 | 35330 | 5.0 (1.9) | Ref. |
| >0-10g/day | 7703 | 5.3 (1.9) | 0.10 (0.04; 0.16) |
| >10-20g/day | 1428 | 5.3 (1.9) | -0.05 (-0.16; 0.06) |
| >20g/day | 373 | 5.3 (2.1) | -0.02 (-0.23; 0.18) |
| **Beer** |  |  |  |
| 0 | 33590 | 5.1 (2.0) | Ref. |
| >0-10g/day | 8866 | 4.9 (1.9) | -0.15 (-0.21; -0.09) |
| >10-20g/day | 1811 | 4.5 (1.9) | -0.24 (-0.35; -0.13) |
| >20g/day | 567 | 4.0 (1.8) | -0.55 (-0.73; -0.36) |
| **Spirits** |  |  |  |
| 0 | 41869 | 5.1 (1.9) | Ref. |
| >0-10g/day | 2213 | 4.3 (1.9) | -0.31 (-0.42 -0.21) |
| >10-20g/day | 618 | 4.1 (1.9) | -0.27 (-0.47; -0.08) |
| >20g/day | 134 | 3.6 (1.9) | -0.54 (-0.90; -0.17) |

^a^ Linear regression model adjusted for sex, age, level of education, size of municipality, country of birth, tobacco consumption, leisure time sedentarism, BMI, perceived health status, survey year, total daily alcohol intake (g/day), and binge drinking.

^b^ Linear regression model adjusted for sex, age, level of education, size of municipality, country of birth, tobacco consumption, leisure time sedentarism, BMI, perceived health status, survey year, alcohol intake (g(day) from other types of alcoholic beverages, and binge drinking.

**Supplementary Table 3. Association between average alcohol consumption and binge drinking, and non-adherence to the Mediterranean diet, excluding proxies.**

|  |  | **Non-Adherence to the Mediterranean Diet** | |
| --- | --- | --- | --- |
|  | **N** | **Prevalence (%)**  **(95%CI)** | **OR**  **(95%CI)** |
|  |  |  |  |
| **Average alcohol consumption^a^** |  |  |  |
| Non-drinkers | 8812 | 73.0 (71.5; 74.4) | Ref. |
| Former drinkers | 6063 | 75.0 (73.5; 76.4) | 1.21 (1.09; 1.35) |
| Occasional drinkers | 12605 | 77.1 (76.0; 78.1) | 1.14 (1.04; 1.25) |
| Low risk | 12261 | 77.9 (77.0; 78.9) | 1.36 (1.23; 1.50) |
| High risk | 3676 | 80.3 (78.6; 81.9) | 1.55 (1.35; 1.79) |
|  |  |  |  |
| **Binge drinking^b^** |  |  |  |
| **In the previous 30 days** |  |  |  |
| No | 40640 | 76.0 (75.3; 76.6) | Ref. |
| Yes | 2777 | 82.9 (81.0; 84.6) | 0.89 (0.78; 1.02) |
|  |  |  |  |
| **Frequency** |  |  |  |
| Never | 36245 | 74.8 (74.1; 75.5) | Ref. |
| <once a month | 4395 | 84.3 (83.0; 85.6) | 1.17 (1.04; 1.31) |
| Monthly | 1980 | 83.1 (81.0; 85.1) | 0.95 (0.81; 1.12) |
| Weekly | 797 | 82.2 (78.7; 85.2) | 0.84 (0.66; 1.06) |

^a^ Logistic regression model adjusted for sex, age, level of education, size of municipality, country of birth, tobacco consumption, leisure time sedentarism, BMI, perceived health status, survey year, and binge drinking after excluding proxies.

^b^ Logistic regression model adjusted for sex, age, level of education, size of municipality, country of birth, tobacco consumption, leisure time sedentarism, BMI, perceived health status, survey year, and total daily alcohol intake (g/day) after excluding proxies.

**Supplementary Table 4. Association between preference and type of alcoholic beverage and non-adherence to the Mediterranean diet, excluding proxies.**

|  |  | **Non-Adherence to the Mediterranean Diet** | |
| --- | --- | --- | --- |
|  | **N** | **Prevalence (%)**  **(95%CI)** | **OR**  **(95%CI)** |
|  |  |  |  |
| **Alcoholic beverage preference^a^** |  |  |  |
| Non-drinkers | 8812 | 73.0 (71.5; 74.4) | Ref. |
| Former drinkers | 6063 | 75.0 (73.5; 76.4) | 1.21 (1.09; 1.35) |
| Occasional drinkers | 12605 | 77.1 (76.0; 78.1) | 1.15 (1.05; 1.26) |
| No preference | 6384 | 79.5 (78.2; 80.7) | 1.29 (1.13; 1.48) |
| Wine | 4049 | 68.4 (66.5; 70.2) | 1.09 (0.96; 1.24) |
| Beer | 5135 | 82.3 (80.9; 83.6) | 1.44 (1.27; 1.65) |
| Spirits | 369 | 89.9 (86.0; 92.8) | 1.87 (1.26; 2.78) |
|  |  |  |  |
| **Type of alcoholic beverage^b^** |  |  |  |
| **Wine** |  |  |  |
| 0 | 34063 | 77.2 (76.4; 77.9) | Ref. |
| >0-10g/day | 7584 | 73.3 (72.0; 74.5) | 0.94 (0.86; 1.02) |
| >10-20g/day | 1400 | 76.2 (73.3; 78.8) | 1.18 (1.00; 1.38) |
| >20g/day | 370 | 72.4 (66.2; 77.8) | 0.98 (0.72; 1.32) |
| **Beer** |  |  |  |
| 0 | 32247 | 74.8 (74.0; 75.5) | Ref. |
| >0-10g/day | 8808 | 79.6 (78.5; 80.7) | 1.26 (1.16; 1.37) |
| >10-20g/day | 1803 | 84.7 (82.4; 86.8) | 1.41 (1.18; 1.69) |
| >20g/day | 559 | 90.2 (87.1; 92.6) | 2.08 (1.48; 2.93) |
| **Spirits** |  |  |  |
| 0 | 40466 | 75.7 (75.0; 76.4) | Ref. |
| >0-10g/day | 2207 | 85.1 (83.3; 86.8) | 1.20 (1.03; 1.39) |
| >10-20g/day | 612 | 86.4 (82.4; 89.5) | 1.15 (0.84; 1.58) |
| >20g/day | 132 | 92.0 (85.8; 95.7) | 1.82 (0.93; 3.57) |

^a^ Logistic regression model adjusted for sex, age, level of education, size of municipality, country of birth, tobacco consumption, leisure time sedentarism, BMI, survey year, and total daily alcohol intake (g/day) after excluding proxies.

^b^ Logistic regression model adjusted for sex, age, level of education, size of municipality, country of birth, tobacco consumption, leisure time sedentarism, BMI, perceived health status, survey year, alcohol intake (g(day) from other types of alcoholic beverages, and binge drinking after excluding proxies.

**Supplementary Table 5. Association between binge drinking, preference, type of alcoholic beverage, and non-adherence to the Mediterranean diet, excluding former drinkers.**

|  |  | **Non-Adherence to the Mediterranean Diet** | |
| --- | --- | --- | --- |
|  | **N** | **Prevalence (%)**  **(95%CI)** | **OR**  **(95%CI)** |
|  |  |  |  |
| **Binge drinking^a^** |  |  |  |
| **In the previous 30 days** |  |  |  |
| No | 35561 | 76.1 (75.3; 76.8) | Ref. |
| Yes | 2801 | 82.9 (81.1; 84.6) | 0.90 (0.78; 1.03) |
|  |  |  |  |
| **Frequency** |  |  |  |
| Never | 31145 | 74.8 (74.0; 75.5) | Ref. |
| <once a month | 4416 | 84.2 (82.9; 85.5) | 1.17 (1.04; 1.31) |
| Monthly | 1991 | 83.2 (81.0; 85.1) | 0.96 (0.81; 1.13) |
| Weekly | 810 | 82.3 (78.9; 85.3) | 0.84 (0.66; 1.07) |
|  |  |  |  |
| **Type of alcoholic beverage^b^** |  |  |  |
| **Wine** |  |  |  |
| 0 | 28858 | 77.4 (76.7; 78.2) | Ref. |
| >0-10g/day | 7703 | 73.4 (72.1; 74.6) | 0.96 (0.89; 1.05) |
| >10-20g/day | 1428 | 76.1 (73.3; 78.7) | 1.20 (1.03; 1.41) |
| >20g/day | 373 | 72.4 (66.2; 77.8) | 1.01 (0.75; 1.37) |
| **Beer** |  |  |  |
| 0 | 27118 | 74.7 (73.9; 75.5) | Ref. |
| >0-10g/day | 8866 | 79.6 (78.5; 80.7) | 1.27 (1.16; 1.38) |
| >10-20g/day | 1811 | 84.7 (82.4; 86.8) | 1.42 (1.19; 1.70) |
| >20g/day | 567 | 90.1 (86.9; 92.5) | 2.08 (1.48; 2.93) |
| **Spirits** |  |  |  |
| 0 | 35397 | 75.8 (75.0; 76.5) | Ref. |
| >0-10g/day | 2213 | 85.1 (83.3; 86.8) | 1.21 (1.04; 1.40) |
| >10-20g/day | 618 | 86.5 (82.6; 89.7) | 1.18 (0.86; 1.63) |
| >20g/day | 134 | 91.8 (85.6; 95.4) | 1.77 (0.92; 3.40) |

^a^ Logistic regression model adjusted for sex, age, level of education, size of municipality, country of birth, tobacco consumption, leisure time sedentarism, BMI, survey year, and total daily alcohol intake (g/day) after excluding former drinkers.

^b^ Logistic regression model adjusted for sex, age, level of education, size of municipality, country of birth, tobacco consumption, leisure time sedentarism, BMI, perceived health status, survey year, alcohol intake (g(day) from other types of alcoholic beverages, and binge drinking after excluding former drinkers.
